# Surgical outcome after spinal fractures in patients with ankylosing spondylitis

**DOI:** 10.1186/1471-2474-10-96

**Published:** 2009-08-02

**Authors:** George Sapkas, Konstantinos Kateros, Stamatios A Papadakis, Spyros Galanakos, Emmanuel Brilakis, George Machairas, Pavlos Katonis

**Affiliations:** 1 Department of Orthopaedics, University of Athens, Attikon University, Hospital, Haidari, Greece; 2 Department of Orthopaedics, University of Athens, Agia Olga General, Hospital, N. Ionia, Greece; 3 Department of Orthopaedics, KAT General Hospital, Kifissia, Greece; 4 Department of Orthopaedics, University of Crete, Herakleion, Greece

## Abstract

**Background:**

Ankylosing spondylitis is a rheumatic disease in which spinal and sacroiliac joints are mainly affected. There is a gradual bone formation in the spinal ligaments and ankylosis of the spinal diarthroses which lead to stiffness of the spine.

The diffuse paraspinal ossification and inflammatory osteitis of advanced Ankylosing spondylitis creates a fused, brittle spine that is susceptible to fracture. The aim of this study is to present the surgical experience of spinal fractures occurring in patients suffering from ankylosing spondylitis and to highlight the difficulties that exist as far as both diagnosis and surgical management are concerned.

**Methods:**

Twenty patients suffering from ankylosing spondylitis were operated due to a spinal fracture. The fracture was located at the cervical spine in 7 cases, at the thoracic spine in 9, at the thoracolumbar junction in 3 and at the lumbar spine in one case. Neurological defects were revealed in 10 patients. In four of them, neurological signs were progressively developed after a time period of 4 to 15 days. The initial radiological study was negative for a spinal fracture in twelve patients. Every patient was assessed at the time of admission and daily until the day of surgery, then postoperatively upon discharge.

**Results:**

Combined anterior and posterior approaches were performed in three patients with only posterior approaches performed on the rest. Spinal fusion was seen in 100% of the cases. No intra-operative complications occurred. There was one case in which superficial wound inflammation occurred. Loosening of posterior screws without loss of stability appeared in two patients with cervical injuries.

Frankel neurological classification was used in order to evaluate the neurological status of the patients. There was statistically significant improvement of Frankel neurological classification between the preoperative and postoperative evaluation. 35% of patients showed improvement due to the operation performed.

**Conclusion:**

The operative treatment of these injuries is useful and effective. It usually succeeds the improvement of the patients' neurological status. Taking into consideration the cardiovascular problems that these patients have, anterior and posterior stabilization aren't always possible. In these cases, posterior approach can be performed and give excellent results, while total operation time, blood loss and other possible complications are decreased.

## Background

Ankylosing Spondylitis (AS) is a chronic inflammatory disease which is characterized by pain and progressive stiffness and which spinal and sacroiliac joints are mainly affected. It affects mostly males, having a male-to-female ratio approximately 3–4:1 and the onset occur between the 15th and the 35th year of life [1-3].

Ankylosing Spondylitis transforms the flexible spinal column into a stiff rod; the stiffened spine cannot bear normal loads in comparison with a healthy spine. In addition, it has been established that bone mineral density (BMD) loss occurs early in the AS disease course and is associated with inflammation correlated with increased bone resorption [4]. The kyphotic deformation of the spine that exists makes the ankylosing and osteoporotic spine susceptible to stress fractures under the impact of small forces and loads [3]. The diffuse paraspinal ossification and inflammatory osteitis of advanced AS creates a fused, brittle spine that is susceptible to fracture [4-8]. Patients suffering from AS may undergo a fracture with minimal [5,9-11] or even no history of injury [12-14].

The most frequent site, where a fracture is located is the cervical spine especially its lower part [5,6,10,12,14-17], and the cervical-thoracic junction, following by the thoracolumbar junction (T10-L2) [9,11-14]. Disruption of all the three columns of the spine predisposes to displacement and neurological injury [4,9,18,19].

When a fracture happens in a patient with AS it should be considered as high-risk injury, especially when it is located in the cervical-thoracic junction of the spine [20,21]. The most unstable types are shearing fractures. They may have severe neurological symptoms or may lead to haemothorax or rupture of the aorta, which are serious complications [21,22]. Secondary neurological aggravation may be possible due to displacement of the fractured segments, which happens mainly in hyperextension injuries [11]. Furthermore, where an interval occurs between trauma and the onset of neurologic signs or worsening of the neurologic picture the formation of an epidural hematoma should be suspected and excluded by means of an MRI scan. [23]. Diagnosis can be difficult due to pre-existing spinal alterations. The standard radiographs are inadequate to fully evaluate shearing fractures due to osteoporosis, and the position of the shoulders (which are usually are located at a higher position). Thus, these fractures can be missed in the first examination and in the later stages, are characterized by vertebral corrosion, collapse and deformity. A misdiagnosed fracture can possibly lead to pseudarthrosis or Andersson lesion [24].

The aim of this study is to present the surgical experience of spinal fractures occurring in patients suffering from AS and to highlight the difficulties that exist as far as both diagnosis and surgical management are concerned.

## Methods

Between 1997 to 2005 twenty patients suffering from AS sustained a spinal fracture and were treated in our department. Their gender, age, the mechanism of injury, the location and the type of the fracture, their neurological status pre and post operatively and their management are reported in table 1. The epidemiological data were obtained from the patient's medical record.

**Table 1:** Summary of patients' data

#	Age (years)	Sex	Mechanism of injury	Level of fracture/Type	Neurological status preoperatively	Treatment/Levels of Fusion	Neurological status postoperatively
1	80	M	Fall	C2/Type II	Frankel C	Posterior instrumentation/Occipito-C4	Frankel D

2	65	M	Fall	C2/Type I	Frankel E	Posterior instrumentation/Occipito-C4	Frankel E

3	60	M	Fall	C6 – C7/A.3.1.1	Frankel E	Anterior + Posterior instrumentation/C4-T2	Frankel E

4	38	M	Fall from height	C6 – C7/A.2.3.1	Frankel C	Anterior + Posterior instrumentation/C4-T2	Frankel E

5	67	M	Fall	C6–C7/B.3.2.2	Frankel C	Posterior instrumentation/C4-T2	Frankel E

6	69	F	Fall	C6 – C7/C.2.2.1	Frankel A	Anterior + Posterior instrumentation/C4-T2	Frankel A

7	55	M	Fall	C6 – C7/A.3.1.1	Frankel E	Posterior instrumentation/C4-T2	Frankel E

8	39	M	Fall	T5 – T6/A.3.3.1	Frankel D	Posterior instrumentation/T3–T8	Frankel E

9	23	F	Fall	T8/A.3.2.3	Frankel E	Posterior instrumentation/T6–T10	Frankel E

10	53	M	Fall	T8 – T9/B.2.2.2	Frankel C	Posterior instrumentation/T6–T11	Frankel D

11	65	F	Fall	T8 – T9/B.1.1.1	Frankel E	Posterior instrumentation/T6–T11	Frankel E

12	57	M	Fall	T9/A.3.2.3	Frankel E	Posterior instrumentation/T7–T11	Frankel E

13	64	M	Fall	T10 – T11/C.2.2.1	Frankel A	Posterior instrumentation/T8-L1	Frankel A

14	79	M	Fall	T10 – T11/A.3.2.1	Frankel B	Posterior instrumentation/T8-L1	Frankel D

15	40	M	Fall	T10 – T11/C.2.1.3	Frankel A	Posterior instrumentation/T8-L1	Frankel A

16	52	M	Car Accident	T11 – T12/B.1.1.1	Frankel E	Posterior instrumentation/T9-L2	Frankel E

17	69	F	Fall	T12 – L1/A.3.2.3	Frankel E	Posterior instrumentation/T10-L2	Frankel E

18	38	M	Fall	T12 – L1/A.3.2.3	Frankel E	Posterior instrumentation/T10-L2	Frankel E

19	40	M	Fall	T12 – L1/B.1.1.1	Frankel E	Posterior instrumentation/T10-L2	Frankel E

20	55	M	Fall	L1 – L2/B.1.1.1	Frankel B	Posterior instrumentation/T12-L4	Frankel D

This is a prospective study, and the patients included were treated operatively. Patients that were treated conservatively were excluded from the study. The surgery was a consideration when the fracture compromised the stability of the spine, when a neurological deficit emerged at the time of diagnosis or during the hospitalisation, or in a combination of the above. Instrumentation spanned four vertebrae, two cephalad and two caudal to the fracture (table 1).

Sixteen out of 20 patients were males with a mean age of 55 years (range, 38–80) and 4 females with a mean age of 56.5 years (range, 23–69). The median time from the point in which the diagnosis was made was 24 years (range, 3–45) (table 2).

**Table 2:** Demographic data and disease characteristics.

**Patients suffered by ankylosing spondylitis who were operated due to a spinal fracture (n = 20)**		
		

Median age (min – max)	56 years (23–80)	

Gender	16 males	80%
	
	4 females	20%

Time from the diagnosis	24 years (3–45)	

		

Cervical spine	7	35%

Thoracic spine	9	45%

Thoraco-lumbar junction	3	15%

Lumbar spine	1	5%

		

Low energy injury	18	90%

High energy injury	2	10%

The fracture was located at the cervical spine in 7 patients, at the thoracic spine in 9, at the thoracolumbar junction in 3, and at the lumbar spine in one patient (table 2). Classification of the spine fractures was made according to AO classification. Clinical examination and radiological imaging defined the level of injury and the neurological status of each patient. Frankel neurological classification was used in order to evaluate patients' neurological status. Each patient was assessed initially at the time of admission, daily until the day of surgery and postoperatively upon discharge. Two experienced orthopaedic surgeons made the clinical examination. The patients were also assessed at 3, 6, 9 and 12 months after the operation and then annually. The average follow up time was 5 years (range, 2–8 years).

Radiographs (anteroposterior, lateral, and oblique views) were performed upon admission. Computed tomography (CT) and Magnetic resonance imaging (MRI) studies were performed in cases where the primary imaging investigation was negative for a fracture but clinical suspicion continued to exist. They were also performed in all of the cases when the operation had already been decided. Two experienced orthopaedic surgeons always assessed the radiological studies.

### Statistical analysis

Data is expressed as mean ± standard deviation for continuous variables and as percentages for categorical data. In order to examine the pre and post operatively difference of Frankel neurological classification, Wilcoxon Signed Ranks Test was used. The McNemar Test would be more appropriate for this but it was not performed because the compared variables were not dichotomous with the same values.

All tests are two-sided and statistical significance was set at p < 0.05. All analyses were carried out using the statistical package SPSS v.13.00 (Statistical Package for the Social Sciences, SPSS Inc., Chicago, Ill., USA).

## Results

In 18 cases, a low energy injury of the spine occurred and in only 2 cases the cause was a high-energy injury (table 2). The fractures occurred in the cervical spine were compression injuries. C6 and C7 levels were the most common fractured vertebrae, followed by C2 vertebra. There was a 43% (three patients) incidence of neurological deficit on initial presentation, while one of them had already established complete paralysis.

The thoracic spinal column was the most susceptible part of the spine for fracture. T8, T9 and T10 were the most common level of fracture, while neurological defect existed in five out of nine patients (56%), with two of them having already established complete paralysis.

**Figure 1:**
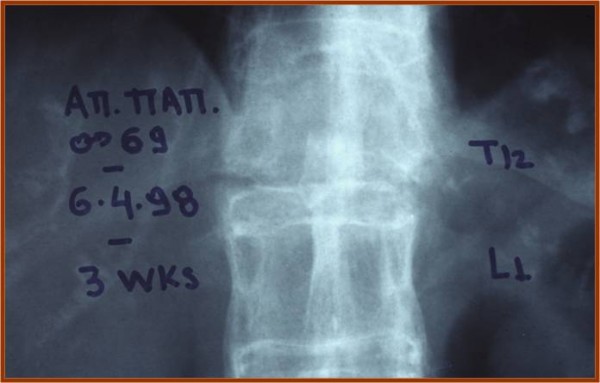
**Chance type fracture due to a hyperextension injury at T12-L1 level with no neurological deficit**.

**Figure 2:**
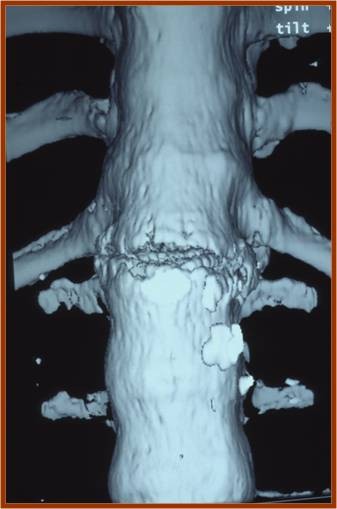
**Anteroposterior 3D reconstruction image of the chance type fracture at T12-L1 level**.

**Figure 3:**
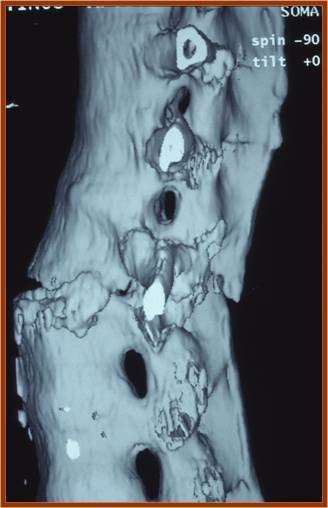
**Lateral 3D reconstruction image of the same case**.

Three patients suffered from fractures of the thoracolumbar junction due to hyperextension injuries without neurological deficit (Figures 1, 2, 3). The lumbar spine was the level of fracture in one patient who had neurological defect on initial presentation. Neurological defects were revealed in ten out of twenty patients. In six of them they were established on initial presentation, while the others established late neurological deficit progressively after a time period of 4 to 15 days.

The initial radiological study was negative for a spinal fracture in 12 patients (60%). In these cases the fracture was revealed by CT or MRI which used in order to investigate the clinical suspicion which existed.

**Figure 4:**
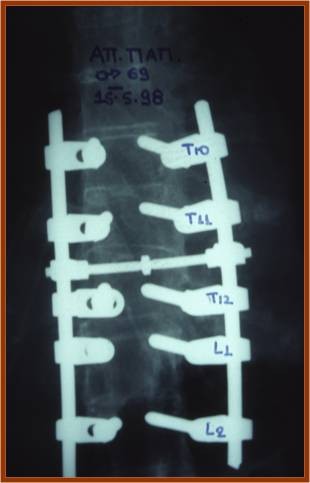
**Anteroposterior postoperative radiograph with a posterior instrumentation system**.

**Figure 5:**
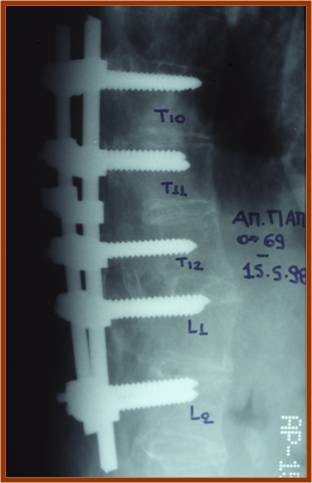
**Lateral postoperative radiograph of the same case**.

As far as treatment is concerned, three of the cervical fractures were managed by a combined approach while the rest were operated on with a posterior approach. A Philadelphia type cervical collar was applied in all patients postoperatively, for 3 to 6 months. In the thoracolumbar spine, a posterior approach was used in all cases (Figures 4, 5). Laminectomy performed in all cases where severe neurological deficit existed. Early mobilization was encouraged and a thoracolumbar spinal orthosis was used for 6–12 months. Fusion was successfully performed in 100% of the cases and it was assessed on the basis of the presence of a homogenous fusion mass on lateral tomographs and/or CT scans.

No intra-operative complications occurred. There was one case in which superficial wound infection occurred. This complication was managed by daily wound changes and the use of local and systematic antibiotics. As far as hardware complications were concerned, loosening of posterior screws without loss of stability appeared in two patients with cervical injuries. In none of the cases epidural hematoma was noted. Spinal deformity was corrected in all of the cases.

The assessment of the patients before the operation according to Frankel neurological classification revealed 3 patients (15%) classified as Frankel A, 2 patients (10%) as Frankel B, 4 patients (20%) as Frankel C, 1 patient (5%) as Frankel D and 10 patients (50%) as Frankel E. After the operation 3 patients (15%) classified as Frankel A, 4 patients as Frankel D (20%) and 13 patients (65%) as Frankel E (table 3).

**Table 3:** Patients' neurological status before and after the operation according to Frankel neurological classification.

**Preoperatively**		Neurological Status	**Postoperatively**	
3	15%	**FRANKEL A**	3	15%

2	10%	**FRANKEL B**	0	0%

4	20%	**FRANKEL C**	0	0%

1	5%	**FRANKEL D**	4	20%

10	50%	**FRANKEL E**	13	65%

Surgical treatment improved patients' neurological status where it had been influenced. The postoperative assessment of the patients was improved, in relation to the preoperative one by at least 1 Frankel grade in 3 patients (15%), while 4 patients (20%) improved by 2 Frankel grades. Three patients (15%) had not any improvement (Frankel A) but had established paraplegia from the initial assessment. Their neurological outcome was poor. The last 10 patients (50%) had no neurological deficit from the time of hospitalization (Frankel E).

According the Wilcoxon Signed Ranks Test which was used, there is statistically significant improvement (p = 0,015) of Frankel neurological classification between the preoperative and postoperative evaluation, once thirty-five per cent of patients presented an improvement (10% from Frankel B to Frankel D, 10% from Frankel C to Frankel D, 10% from Frankel C to Frankel E and 5% from Frankel D to Frankel E) while 65% of patients were in stable condition (15% from Frankel A to Frankel A and 50% from Frankel E to Frankel E) (table 4).

**Table 4:** Comparison between the neurological status of patients suffered by ankylosing spondylitis before and after the operation due to spinal injury, using the Frankel neurological classification.

			Postoperatively		
	
	**Frankel**		**A**	**D**	**E**
Preoperatively	**A**	N	3	-	-
		
		%	15%	0%	0%
	
	**B**	N	-	**2**	-
		
		%	0%	**10%**	0%
	
	**C**	N	-	**2**	**2**
		
		%	0%	**10%**	**10%**
	
	**D**	N	-	-	**1**
		
		%	0%	0%	**5%**
	
	**E**	N	-	-	10
		
		%	0%	0%	50%

***Wilcoxon Signed Ranks Test: p = 0,015***					

## Discussion

In the patients with AS when a fracture occurs, the spine tends to be displaced in hyperextension, especially when the patient is in supine position [13]. This hyperextension may be the main cause of secondary neurological impairment [6,13]. This is the possible explanation for the late neurological complications after a long period of immobilization [6].

In this study, ten out of twenty patients had neurological deficit and four of them obtained late neurological impairment, which was established progressively after a time period of four to fifteen days. The high clinical suspicion is the first diagnostic tool for the otrhopaedics surgeon in order to identify the bone injury, since the severity of the injury is frequently very low [9-11].

All available radiological tools should be used in order to validate the diagnosis, particularly when the injury concerns the occipital-cervical, the cervical-thoracic, the thoracolumbar or the lumbar-sacral junctions. X-rays (anteroposterior, lateral and oblique views) of the injured region may not reveal the fracture, giving only indirect information, such as widening of the disk space and discontinuity of the ossified paraspinal ligaments which isn't able to set the diagnosis [5].

In our study, the initial radiological study was negative for a spinal fracture in twelve out of twenty patients (60%). Where standard radiographs are inadequate, computed tomography can be useful and should be resorted to. The use of CT scanning and MRI scanning has been shown to increase the sensitivity of initial radiographic assessment. Magnetic Resonance Imaging scans are very sensitive in picking up soft tissue injuries and in this group of patients in identifying the presence of epidural hematomas. However, MRI cannot be recommended as a first line investigation in the patient with AS, but may add important information in difficult cases. [23]

The above imaging techniques (CT and MRI) offer valuable help in revealing the type of fracture. This definition is important because the stability of the spine, the management of the injury and the possible complications are related to the type of fracture.

Conservative treatment either by prolonged bed rest in traction or in a cervical collar, or by early realignment and immobilization in a halo vest has been advocated because of supposed higher mortality after surgery [9]. However, maintaining reduction is a major concern for conservative treatment: distraction, halo vest application, and transfer to a stretcher have led to secondary dislocation and neurological deterioration. Furthermore, immobilization in a halo has been associated with serious complications. Poor bone quality, vulnerable skin, and difficulty in achieving good alignment are additional arguments against the use of a halo [25].

It is generally assumed that the stabilization of cervical fractures is better performed with anterior and posterior support of the spine. In this study, three of the cervical fractures were managed by both anterior and posterior approaches while all the rest were managed only by posterior approach, having no intra-operative complications, but one case with superficial wound infection and two cases (patients with cervical injuries) with loosening of posterior screws without loss of stability.

Olerud et al. [13] believe that in the cervical spine, where implant loosening is a considerable problem, the failure of support is presented mainly in cases where only anterior or only posterior stabilization was applied because the stabilizing system may not be able to confront the forces which act on it. Thus, both anterior and posterior stabilization of the spine should be applied, especially for the cervical and the thoraco-lumbar spine. Nevertheless, in everyday practice posterior stabilization is usually performed. This is in order to reduce the possible causal factors of intra-operative and postoperative complications, taking into consideration that the most of these patients have cardiovascular and pulmonary disorders caused by restrictive ankylosis of the thoracic cage and prolonging the operating time by performing double stabilization and thoracotomy aggravates cardiovascular function. Moreover, the anterior approach to the cervical-thoracic junction is extremely difficult in these patients due to the great inclination and the kyphosis that exists at this region.

Long stabilizing systems that offer support to a greater area of the spine and the parallel use of braces postoperatively have been used in order to strengthen the stabilization. Serin et al. [26] showed that four levels posterior fixation is superior to two levels posterior fixation and a four levels fixation plus offset hook is the most stable. Tezeren and Kuru [27] demonstrated that final outcome regarding sagittal index and anterior body compression is better in the long segment instrumentation group than in the short segment instrumentation group.

The percentages of complications and mortality are high. Murray and Persellen [28], refer that the mortality rate of patients who undergo an early operation fluctuates between 15% and 50%. Moreover, patients managed conservatively have a high mortality rate equal to 25% [29]. It is widely accepted that operative treatment should be considered when the spine is unstable or when there is neurological impairment [9,23,30].

In our series no epidural hematomas were noted. In the literature there is some controversy regarding the best way of managing this complication, reports describing good outcome following both surgical and conservative management [31,32]. There is a need for wider multicenter studies to get a correct picture of the incidence and the problems encountered in management of vertebral column trauma in AS.

## Conclusion

Even minor injuries may cause fractures in an ankylosing spine. Patients with AS who sustain injuries of the spine are at greater risk of developing neurological impairment. These neurological disorders may be established at the time of injury but it is not unusual for them to become progressively, with several days delay. It is not an exaggeration to say that new back pain in patients with AS should be assumed to be caused by a fracture until proven otherwise. Thus, thorough clinical and radiological assessment should be performed in these patients and should be repeated for the first few weeks, especially if the patient complains of indefinable pain or if neurological disorders are noted. Accident and Emergency physicians should always bear in mind that simple radiological evaluation of these injuries may not be able to reveal fractures at first. CT and MRI are valuable tools in order to reveal these fractures.

The operative treatment of these injuries is useful and effective for these patients. It usually succeeds the improvement of the patients' neurological status, apart from cases where paraplegia is already established. However, the operative treatment is very demanding, especially when the cervical spine is concerned. Both anterior and posterior stabilization offer better support. Taking into consideration the cardiovascular and pulmonary problems that these patients have, anterior and posterior stabilization aren't always possible.

## Competing interests

The authors declare that they have no competing interests.

## Authors' contributions

GS, KK, SAP and PK participated in the design of the study, data acquisition and analysis and writing of this manuscript. SG and EB participated in the analysis and writing of this paper. KK, SAP and GM participated in the analysis and also in revising critically the manuscript. All authors read and approved the final manuscript.

## Pre-publication history

The pre-publication history for this paper can be accessed here:


